# Nervousness in Soldiers

**Published:** 1918-08

**Authors:** Foster Kennedy


### WAR NEUROSES

**On the Nature of Nervousness in Soldiers.** Major Foster Kennedy, M. D., F. R. S. (Edin.) Major, R. A. M. C., presented a paper in which he said that for eighteen months the term " shell-shock " has been employed in medical literature and colloquially in the British Army, to cover all cases of nervous instability occurring in the course of war. Under this heading have been massed cases of amnesia, anergic stupor, sleeplessness, nightmare, mutism, functional blindness, tremors, palsies, and further, anxiety neuroses, occurring not only under fighting strain but in individuals who — failing in self-confidence — suffer doubts and apprehensions while still waiting for transport overseas. Further, the term became commonly used in newspaper journalism, whence it passed into common speech, always associated with a train of thought in the mind of the speaker, at once fearful and mysterious.

The general acceptance of this term and the apparent recognition by both medical officers and the public of a concrete and, above all, quite novel condition induced by experiences of unimaginable horror — the " moving accidents of flood and field " — had the obvious result of satisfying many a patient as to the propriety of his ailment; there was inevitably induced in him an easy rationalization of his illness, an easy self-justification in the very label tied to his tunic in the field ambulance, which, in all but the most redoubtable spirits, facilitated acceptance of his fate and to some extent inhibited effort on his own part toward his own cure.

A conscious assumption of symptoms by soldiers is rare; it is *not* rare for a man to go sick for a few hours to obtain a temporary alleviation of his lot, but very seldom does one meet a man malingering with a view to discharge from service.

The speaker said that much has been written on the theory that the neuroses of war are the result of direct aerial concussion produced by the explosions of heavy shells. Brains have been sectioned belonging to soldiers alleged to have been killed by "commotio cerebri" resulting from such concussion, and petechial hemorrhages have been demonstrated in such brains in the medulla, midbrain, and basal ganglia. One may submit the great difficulty of obtaining accurate details as to the circumstances of death on the field. It is seldom easy to learn whether or not the man killed was buried and asphyxiated though not outwardly wounded; and one should also bear in mind the power of a heavy shell explosion to deoxygenate the air in its vicinity, before coming overrapidly to the conclusion that only the obvious factors of concussion and compression — even to the extent of ten tons to the square yard — are the sole destroying agents involved in the question.

*Almost never are generalized psychoneuroses seen in soldiers suffering also from physical wounds.*

It is not conceivable that, of two men exposed in equal proximity to a heavy shell burst, he who has no injury can have sustained a greater concussion than he who, for example, has also suffered a transverse lesion of his spinal cord; yet the speaker said he had never seen any sign of nervous instability, — not to say stupor, amnesia, functional abolition of the special senses, or tic, — in any individual at the same time dangerously wounded.

The emotions of fear and pain constitute together our machinery of self-preservation; in most of us, swathed in the cotton-bandages of our civilized lives, but little call is made upon them. Constant exposure to imminent destruction in war produces, however, a tautness of the nervous system, a strain due to powerful excitement and, — as the speaker said — to the organic stress induced by the mobilization of biological instincts heretofore dormant. These instincts of self-preservation do not always — and perhaps not often, — become conscious realizations. The speaker means that men, though in great danger, quite honestly may not feel afraid, their nervous systems may be said to be frightened but their awareness knows no fear.

This submersion of a powerful emotive force below the threshold of consciousness is due partly, perhaps, to the knowledge of the individual of the debilitating effects on his energies of the entrance of fear into his conscious life, but much more, one feels sure, to the inhibitory influence of his morale. Now what is this thing we call morale? — Is it not the expression in each soldier of his herd-instinct, of his willingness to sacrifice himself for the benefit of his kind, and for the ideals held in common by his country-men and himself? It is a loyalty to his mates, to his officers, to his regiment, to his nation, and, in the last instance, to the ideals of life for which his nation stands, and it is measured by his conscious willingness to suffer, his capacity for sacrifice in the common good. It is a quality born of the tribe, a product of gregariousness and so held socially in good repute. It is constantly expressed in thought — it is a real component of the soldiers conscious intellectual life. The shrinking from loss and the fear of death on the other hand are but rarely scrutinized in their realities; they are anti-social in trend and so are cast, by good citizens, into the limbo of subsconciousness.

The speaker's observations that generalized psycho-neuroses do not coexist with severe somatic injury may not be the opinion of all observers, but such observers are speaking of cases seen after transfer to England, who on, and near the field, exhibited no evidences of nervous unrest. In other words, the neurotic symptoms in such cases developed after the lapse of an interval of days or weeks from the date of injury, which proves conclusively the psychogenetic character of these symptoms; and also the uncomfortable fact that unwise suggestion from medical officers can do much to evolve and perpetuate somatic symptoms of psychic origin.

Further, the subject is confused by inadequate classification. The speaker said he had used the term "generalized psycho-neurosis" to include those patients in which inability "to carry on" is the result of their mental and emotional conflicts that have been decided against their higher selves, whose morale has given way before the aggrandizement of their emotions of self-preservation; these are the tremblers, the amnesic, the disoriented, those with night and day dream-deliria, the stuporous; the anxiety-neuroses are a milder type of the same great category, are most often developed in officers, and result from prolonged strain and mental conflict rather than from a single external catastrophe.

Associated with the above symptoms, or more often occurring independently, are various losses or perversions of localized function, usually classed as hysterical stigmata. These may persist after the patient has superficially resumed normal emotional control. On the whole, however, mutism, deafness, functional monoplegia, paraplegia and functional spasms of the limbs are the result of *localized suggestion* rather than of a generalized overwhelming of all the mental and emotional qualities, producing automatism and the temporary replacement of volitional by instinctive life.

By " localized suggestion " is meant some circumstances calculated to produce in a mind, already apprehensive and strained, a more or less fixed idea of localized injury.

The perversions, and abrogations of function due to localized suggestion do not differ essentially from those seen in all neurological hospitals of civil life. Naturally, the suggestions producing these conditions are of the most varied character and identical disabilities may occur through a minor injury from being struck by flying earth or, as a result of a more serious injury, from the diagnostic or verbal indiscretions of a medical officer.

Almost all injuries of the extremities are accompanied for a short period, at least, by a natural " defence-immobilization " of the limb quite apart from any organic nerve injury. This is a natural reaction from pain in tired individuals — a flesh wound of the upper arm, for instance, easily produces in such men the impression of inability to move the wrist and hand, — and any doubts expressed by a doctor as to whether or not, for example, a brachial plexus injury might not possibly account for the condition of the hand, sow the seeds of a fixed idea which later may assume a very sturdy growth.

This brings us to the vital factor underlying the successful treatment of all somatic expressions of psychical unrest — in a word it is *accurate technical knowledge*, — the power to make a careful physical examination, to weigh evidence with precision and, thereby, to attain correct diagnosis : this is the only power which will give the medical officer sufficient self-confidence to enable him to communicate healing to his patient.

Time and again has one seen a half-doubt in diagnosis prevent a coming together of the minds of doctor and patient : if the medical officer be not *quite* sure of the nature of the condition under review, his ability to cure it will be inhibited by a suspicion of the existence either of an organic nerve lesion, on the one hand, or of a conscious malingering, on the other.

In short, functional motor and sensory palsies, and functional perversions of the special senses are created by suggestion directed towards the affected faculty or member, and are susceptible of cure by like means and by like means only. To differentiate them easily and rapidly from similarly appearing organic conditions is the first and most important step in their treatment, and one which, when taken with firmness and accuracy, will confer on the medical officer the self-confidence and authority to exorcise the system of false ideas which has been the immediate cause of the condition.

We are indebted to Freud and his school for our realization that neurotic symptoms may be produced by the antagonism of mutually incompatible emotional trends. The tremendous mass of material made available by war demonstrates the general rightness of this principle, but still more definitely proves the peculiar wrongness of the details with which the psycho-analysts have applied it, and entirely invalidates their deduction — elaborated as a pontifical dogma — that the sexual instinct in various disguises is the only dynamic force possibly concerned.

Soldiers since the beginning of armies have been clear-eyed in this knowledge. They have not, of course, dealt in the turgid phrases of our schools, but they have felt surely the binding qualities of the herd, and have seen in them the only emotive force fit to overcome in their men the fear of death. They too have known that the cement of the herd is the suggestibility of man and their instrument of suggestion is called discipline.

So in this matter the medical officer has an onerous and difficult task. He acts both for the state and for the soldier and, to deal fairly with both clients, it is vital that he should appreciate the truly psychological character of the problem with which he has to wrestle. Only by so doing can he free his patient of his symptoms and, what is still more important, protect the armies from the contagion of suggestion so apt to sweep through such closely co-ordinated communities, each individual of which is exposed to identical causative conditions.

The essential nature of these ailments having been grasped, they must be classified and christened. The term " shell shock " founded on false premises, has served not only to suggest an incorrect etiology, but, by its pitiful and romantic sound, has tended to perpetuate symptoms and to excite no determination in the mind of the sufferer to recover his control, or, in the fighting man, still to endure. So far is it from making an appeal to conscience or to discipline, that it stifles both, and stultifies effort towards cure.

The name is a mistake : we must be rid of it. Let us have instead a true term which will be neither a compromise nor a technicality, unintelligible to the mind of the soldier.

Hysteria is unsuitable in that its significance to laymen and doctors is not identical, nor does it embrace, for instance, such conditions as the anxiety-neuroses. The simple word " nervousness " comprises all the neurotic manifestations seen in war. It furnishes an appeal to the sense of discipline in the armies, and further promotes the growth of a public opinion, both military and civil, which would be of the greatest prophylatic and therapeutic power.

This diagnosis would continue to be divided into nervousness (sick) and nervousness (wounded), as now obtains, according to the external conditions to which the man was exposed at the time of break down. This change in military nomenclature would make clear to both soldiers and civilians that such diagnoses need not necessarily be followed by a return to home or to the Base, and would clearly indicate the propriety of dealing with such cases diagnostically and therapeutically in rest camps, and especially work camps, in front areas and on the lines of communication.

The author stated he had only attempted to give in general terms the nature of the problem with which we are confronted. In doing so, only the psychological aspects were regarded, because these mental symptoms can be examined easily, and treated successfully — though empirically — by rest, persuasion, re-education and the restoration of self-confidence through suggestion and discipline. He was not unmindful, however, of the physical changes which, accompanying violent emotional disturbances, result in alterations in the physiological balance of the involuntary nervous system. One cannot yet know whether such reflex and secretory activities are the *cause* or the *result* of mental and emotional stress.

The collection of clinical and experimental data on such phenomena as the functional disorders of cardiac rhythm following emotional tension — or dyspnea accompanied by increased absolute blood acidity, and similar cognate problems may, in the future, throw light on the association or integrative activities of mind and body.

Such data, however, do little, as yet, to reveal the intrinsic nature of mind. In the meantime, to cope adequately with the wastage of health and of armies involved in this question, we must perforce deal with mental phenomena rather than with mind, and apply, with some empiricism, psychological remedies to psychological ills.

